# Supplementary material for: The Potential Connection between Molecular Changes and Biomarkers Related to ALS and the Development and Regeneration of CNS
Source: Int J Mol Sci. 2022 Sep 26;23(19):11360. doi: 10.3390/ijms231911360 (PMC9570269; doi:10.3390/ijms231911360)
Supplement: Supplementary file 1 [file ijms-23-11360-s001.zip › ijms-1859722-supplementary.pdf]

Supplementary Table S1. ALS - marsupials - related genes from Table 2 and their up to five most predicted interacting human miRNA and lncRNA molecules, indicating their expression in the blood, muscles and spinal cord.

Genes		miRNA (MIRT)	miRNA	miRNA expression
ENSEMBL	Name	lncRNA (ENST)	lncRNA	lncRNA expression
ENSG00000145901	TNIP1 / NAF1	MIRT024151	hsa-miR-221-3p	B+, S+, C+
B+++, S+++, C+++		MIRT042899	hsa-miR-324-3p	B+, S++, C+
		MIRT047340	hsa-miR-181a-5p	B+, S+
		MIRT438115	hsa-miR-517a-3p	B
		MIRT518704	hsa-miR-1277-5p	B
		ENST00000627551	RP11-573D15.8-018	
		ENST00000623959	RP3-323A16.1-001	
		ENST00000527021	AP006621.9-001	
		ENST00000623111	MIR6820-001	
		ENST00000601203	AIRN-001	
ENSG00000163808	KIF15	MIRT016562	hsa-miR-193b-3p	B, S
B++, S++, C++		MIRT024287	hsa-miR-215-5p	B+, C
		MIRT026795	hsa-miR-192-5p	B+, S+, C+
		MIRT440373	hsa-miR-218-5p	S+, C+
		ENST00000627551	RP11-573D15.8-018	
		ENST00000623195	RP11-340L3.1-001	
		ENST00000529733	MIR100HG-008	
		ENST00000527474	MIR100HG-001	
		ENST00000623027	CTA-992D9.11-001	
ENSG00000070761	CFAP20	MIRT790090	hsa-miR-129-5p	B
B++, S++, C+++		MIRT790336	hsa-miR-3680-3p	B+, S+
		MIRT790421	hsa-miR-4307	
		MIRT790505	hsa-miR-450b-5p	S+
		MIRT790562	hsa-miR-4691-5p	B+, S+, C+

		ENST00000627551	RP11-573D15.8-018	
		ENST00000589281	RP11-95O2.5-001	
		ENST00000527021	AP006621.9-001	
		ENST00000564672	CTB-134F13.1-001	
		ENST00000553344	CTD-3051D23.4-001	
ENSG00000103051	COG4	MIRT039829	hsa-miR-615-3p	B, S+
B++, S++, C++		MIRT040817	hsa-miR-18a-3p	B
		ENST00000627551	RP11-573D15.8-018	
		ENST00000392630	C10orf91-001	
		ENST00000527021	AP006621.9-001	
		ENST00000607453	RP11-156E8.1-001	
		ENST00000623111	MIR6820-001	
ENSG00000183735	TBK1	MIRT003360	hsa-miR-221-3p	B+, S+, C+
B++, S++, C++		MIRT006971	hsa-miR-200c-3p	B
		MIRT053453	hsa-miR-452-5p	S+
		MIRT066413	hsa-let-7f-2-3p	B
		MIRT066414	hsa-miR-1185-2-3p	
		ENST00000610945	RP11-114F3.4-001	
		ENST00000419895	RP11-356J5.12-001	
		ENST00000566699	JHDM1D-AS1-001	
		ENST00000534909	OTX2-AS1-001	
		ENST00000399869	RP11-43F13.3-001	
ENSG00000130477	UNC13A	MIRT029920	hsa-miR-26b-5p	B+
B+, S+, C++		MIRT439341	hsa-miR-541-5p	S+
		MIRT439342	hsa-miR-432-5p	
		MIRT439343	hsa-miR-409-3p	B
		MIRT464409	hsa-miR-1260b	B+, S+, C+
		ENST00000627551	RP11-573D15.8-018	
		ENST00000623111	MIR6820-001	

		ENST00000623959	RP3-323A16.1-001	
		ENST00000626826	HELLPAR-001	
		ENST00000339021	LA16c-358B7.4-001	
ENSG00000166685	COG1	MIRT042242	hsa-miR-484	B+
B++, S++, C++		MIRT045480	hsa-miR-149-5p	S+, C+
		ENST00000627551	RP11-573D15.8-018	
		ENST00000527021	AP006621.9-001	
		ENST00000623959	RP3-323A16.1-001	
		ENST00000607453	RP11-156E8.1-001	
		ENST00000589281	RP11-95O2.5-001	
ENSG00000135775	COG2	MIRT020037	hsa-miR-375	B
B++, S++, C++		MIRT021018	hsa-miR-155-5p	B
		MIRT045156	hsa-miR-186-5p	B+, S+, C+
		ENST00000627551	RP11-573D15.8-018	
		ENST00000527021	AP006621.9-001	
		ENST00000589281	RP11-95O2.5-001	
		ENST00000607453	RP11-156E8.1-001	
		ENST00000573167	BAIAP2-AS1-002	
ENSG00000164597	COG5	MIRT027656	hsa-miR-98-5p	S+
B++, S++, C++		MIRT044640	hsa-miR-320a	B+, S+
		MIRT538432	hsa-miR-3978	B, S+
		MIRT538433	hsa-miR-4261	B
		MIRT538434	hsa-miR-222-3p	B+
		ENST00000627551	RP11-573D15.8-018	
		ENST00000432442	GS1-519E5.1-001	
		ENST00000623959	RP3-323A16.1-001	
		ENST00000623111	MIR6820-001	
		ENST00000623027	CTA-992D9.11-001	
ENSG00000168434	COG7	MIRT518036	hsa-miR-6807-5p	

B++, S++, C++		MIRT518037	hsa-miR-7151-3p	
		MIRT518038	hsa-miR-5095	
		MIRT518039	hsa-miR-6504-3p	
		MIRT518040	hsa-miR-4438	
		ENST00000627551	RP11-573D15.8-018	
		ENST00000623111	MIR6820-001	
		ENST00000623726	RP5-1014D13.2-001	
		ENST00000624264	CTA-212A2.3-001	
		ENST00000623075	CTA-941F9.10-001	
ENSG00000196090	PTPRT	MIRT017080	hsa-miR-335-5p	
B+, S+, C++		MIRT043524	hsa-miR-331-3p	B+, S+, C+
		MIRT047447	hsa-miR-10b-5p	S+
		MIRT047550	hsa-miR-10a-5p	B+, S+, C+
		MIRT051085	hsa-miR-16-5p	B+
		ENST00000627551	RP11-573D15.8-018	
		ENST00000527021	AP006621.9-001	
		ENST00000623959	RP3-323A16.1-001	
		ENST00000589281	RP11-95O2.5-001	
		ENST00000623111	MIR6820-001	
ENSG00000054356	PTPRN	MIRT040943	hsa-miR-18a-3p	B
B+, S+, C++		ENST00000627551	RP11-573D15.8-018	
		ENST00000623111	MIR6820-001	
		ENST00000623959	RP3-323A16.1-001	
		ENST00000607453	RP11-156E8.1-001	
		ENST00000597346	KCNQ1OT1-001	
ENSG00000173482	PTPRM	MIRT018602	hsa-miR-335-5p	
B+, S++, C++		MIRT054104	hsa-miR-205-5p	B
		MIRT654607	hsa-miR-3680-3p	B+, S+
		MIRT654608	hsa-miR-557	B

		MIRT654609	hsa-miR-507	B
		ENST00000627551	RP11-573D15.8-018	
		ENST00000527021	AP006621.9-001	
		ENST00000623959	RP3-323A16.1-001	
		ENST00000607453	RP11-156E8.1-001	
		ENST00000589281	RP11-95O2.5-001	
ENSG00000092108	SCFD1	MIRT044947	hsa-miR-186-5p	B+, S+, C+
B++, S++, C++		ENST00000627551	RP11-573D15.8-018	
		ENST00000603191	RP11-230C9.2-001	
		ENST00000414504	ATXN8OS-002	
		ENST00000413911	AC005540.3-001	
		ENST00000418006	LINC00940-001	
ENSG00000168028	RPSA	MIRT031978	hsa-miR-16-5p	B+
B+++, S+++, C+++		MIRT036398	hsa-miR-1227-3p	B+, S+, C+
		MIRT039076	hsa-miR-769-3p	B+, C
		MIRT039727	hsa-miR-615-3p	B, S+
		MIRT045843	hsa-miR-132-3p	B, C
		ENST00000627551	RP11-573D15.8-018	
		ENST00000425966	AC018462.2-001	
		ENST00000421323	AC018462.2-002	
		ENST00000602812	FTX-009	
		ENST00000623959	RP3-323A16.1-001	
ENSG00000125991	ERGIC3	MIRT007115	hsa-miR-490-3p	B+, C+
B++, S++, C++		MIRT032333	hsa-let-7b-5p	B+, S+, C+
		ENST00000627551	RP11-573D15.8-018	
		ENST00000623111	MIR6820-001	
		ENST00000623959	RP3-323A16.1-001	
		ENST00000597346	KCNQ1OT1-001	
		ENST00000601203	AIRN-001	

ENSG00000089280	FUS	MIRT004196	hsa-miR-197-3p	
B++, S++, C++		MIRT035826	hsa-miR-664a-3p	B, C
		MIRT036115	hsa-miR-1296-5p	B+, C
		MIRT040469	hsa-miR-615-3p	B, S+,
		MIRT043384	hsa-miR-331-3p	B+, S+,C+
		ENST00000361558	MUC2-001	
		ENST00000613187	MUC2-201	
		ENST00000623111	MIR6820-001	
		ENST00000623959	RP3-323A16.1-001	
		ENST00000626826	HELLPAR-001	
ENSG00000054523	KIF1B	MIRT016600	hsa-miR-193b-3p	B, S
B++, S++, C++		MIRT021443	hsa-miR-9-5p	B, C+
		MIRT023106	hsa-miR-124-3p	B
		MIRT030116	hsa-miR-26b-5p	B+
		MIRT031988	hsa-miR-16-5p	B+
		ENST00000627551	RP11-573D15.8-018	
		ENST00000623111	MIR6820-001	
		ENST00000623959	RP3-323A16.1-001	
		ENST00000624945	RP3-394A18.1-001	
		ENST00000623726	RP5-1014D13.2-001	
ENSG00000168280	KIF5C	MIRT024749	hsa-miR-215-5p	B+, C
B+, S+, C+++		MIRT026185	hsa-miR-192-5p	B+, S+, C+
		MIRT044274	hsa-miR-106b-5p	B+, S+, C
		MIRT046723	hsa-miR-222-3p	B+
		MIRT047105	hsa-miR-183-5p	B
		ENST00000627551	RP11-573D15.8-018	
		ENST00000548231	RP11-210M15.1-001	
		ENST00000597346	KCNQ1OT1-001	
		ENST00000626826	HELLPAR-001	

		ENST00000623027	CTA-992D9.11-001	
ENSG00000174996	KLC2	MIRT042175	hsa-miR-484	B+
B++, S++, C++		MIRT043375	hsa-miR-331-3p	B+, S+, C+
		MIRT047944	hsa-miR-30c-5p	B
		MIRT049334	hsa-miR-92a-3p	B
		MIRT727346	hsa-miR-195-5p	B
		ENST00000627551	RP11-573D15.8-018	
		ENST00000623111	MIR6820-001	
		ENST00000623959	RP3-323A16.1-001	
		ENST00000597346	KCNQ1OT1-001	
		ENST00000623130	AC006548.28-001	
ENSG00000137171	KLC4	MIRT018861	hsa-miR-335-5p	
B++, S++, C++		MIRT035911	hsa-miR-1180-3p	B, C
		ENST00000627551	RP11-573D15.8-018	
		ENST00000623111	MIR6820-001	
		ENST00000339021	LA16c-358B7.4-001	
		ENST00000623959	RP3-323A16.1-001	
		ENST00000607453	RP11-156E8.1-001	
ENSG00000116852	KIF21B	MIRT029731	hsa-miR-26b-5p	B+
B++, S+, C++		MIRT440370	hsa-miR-218-5p	B, S+, C+
		MIRT489185	hsa-miR-484	B+
		MIRT489186	hsa-miR-6874-5p	
		MIRT489187	hsa-miR-4633-3p	B, C
		ENST00000627551	RP11-573D15.8-018	
		ENST00000623111	MIR6820-001	
		ENST00000623959	RP3-323A16.1-001	
		ENST00000597346	KCNQ1OT1-001	
		ENST00000626826	HELLPAR-001	
ENSG00000142168	SOD1	MIRT000992	hsa-miR-377-3p	B

B++, S+++, C+++		MIRT043913	hsa-miR-378a-3p	B, S, C
		MIRT048056	hsa-miR-197-3p	
		MIRT735173	hsa-miR-206	B, S+
		ENST00000623195	RP11-340L3.1-001	
		ENST00000623027	CTA-992D9.11-001	
		ENST00000419895	RP11-356J5.12-001	
		ENST00000600671	CTB-176F20.3-001	
		ENST00000528381	MIR100HG-007	
ENSG00000178950	GAK	MIRT001367	hsa-miR-1-3p	B, S
B++, S++, C++		MIRT037433	hsa-miR-744-5p	B+, S+, C+
		MIRT044214	hsa-miR-301a-3p	B, S+, C+
		MIRT049506	hsa-miR-92a-3p	B
		MIRT440590	hsa-miR-20a-5p	
		ENST00000627551	RP11-573D15.8-018	
		ENST00000527021	AP006621.9-001	
		ENST00000623959	RP3-323A16.1-001	
		ENST00000607453	RP11-156E8.1-001	
		ENST00000432442	GS1-519E5.1-001	
ENSG00000123240	OPTN	MIRT019128	hsa-miR-335-5p	
B++, S+++, C++		MIRT021455	hsa-miR-9-5p	B, C+
		MIRT049818	hsa-miR-92a-3p	B
		MIRT050771	hsa-miR-17-3p	
		MIRT050845	hsa-miR-17-5p	
		ENST00000623111	MIR6820-001	
		ENST00000626826	HELLPAR-001	
		ENST00000627551	RP11-573D15.8-018	
		ENST00000623075	CTA-941F9.10-001	
		ENST00000623959	RP3-323A16.1-001	
ENSG00000130294	KIF1A	MIRT038023	hsa-miR-423-5p	B, C

B+, S+, C+++		MIRT039561	hsa-miR-652-3p	B
		MIRT042597	hsa-miR-423-3p	B, C
		MIRT045431	hsa-miR-149-5p	B, S+, C+
		MIRT045783	hsa-miR-9-5p	B, C+
		ENST00000627551	RP11-573D15.8-018	
		ENST00000623111	MIR6820-001	
		ENST00000626826	HELLPAR-001	
		ENST00000623959	RP3-323A16.1-001	
		ENST00000623130	AC006548.28-001	
ENSG00000068796	KIF2A	MIRT003853	hsa-miR-1-3p	B, S
B++, S++, C++		MIRT004016	hsa-miR-183-5p	B
		MIRT025350	hsa-miR-34a-5p	B+, S+, C+
		MIRT031888	hsa-miR-16-5p	B+
		MIRT032432	hsa-let-7b-5p	B+, S+, C+
		ENST00000627551	RP11-573D15.8-018	
		ENST00000623111	MIR6820-001	
		ENST00000597346	KCNQ1OT1-001	
		ENST00000623959	RP3-323A16.1-001	
		ENST00000623130	AC006548.28-001	
ENSG00000131437	KIF3A	MIRT440368	hsa-miR-155-5p	B
B+, S++, C++		MIRT474702	hsa-miR-3915	S+, C
		MIRT474703	hsa-miR-1294	B+, S+
		MIRT474704	hsa-miR-4316	B
		MIRT474705	hsa-miR-3928-3p	B, C
		ENST00000623111	MIR6820-001	
		ENST00000626826	HELLPAR-001	
		ENST00000623959	RP3-323A16.1-001	
		ENST00000623726	RP5-1014D13.2-001	
		ENST00000624072	CTA-228A9.4-001	

ENSG00000101350	KIF3B	MIRT043232	hsa-miR-324-5p	B+, S++, C+
B++, S++, C++		MIRT046602	hsa-miR-222-3p	B+
		MIRT047522	hsa-miR-10a-5p	B+, S+, C+
		MIRT152736	hsa-miR-15a-5p	B
		MIRT152737	hsa-miR-16-5p	B+
		ENST00000626826	HELLPAR-001	
		ENST00000623111	MIR6820-001	
		ENST00000623130	AC006548.28-001	
		ENST00000624628	RP13-580B18.4-001	
		ENST00000623959	RP3-323A16.1-001	
ENSG00000170759	KIF5B	MIRT021905	hsa-miR-128-3p	B, S+, C+
B++, S++, C+++		MIRT023550	hsa-miR-1-3p	B, S
		MIRT024776	hsa-miR-215-5p	B+, C
		MIRT025237	hsa-miR-34a-5p	B+, S+, C+
		MIRT026150	hsa-miR-192-5p	B+, S+, C+
		ENST00000627551	RP11-573D15.8-018	
		ENST00000432442	GS1-519E5.1-001	
		ENST00000607453	RP11-156E8.1-001	
		ENST00000527021	AP006621.9-001	
		ENST00000623959	RP3-323A16.1-001	
ENSG00000075945	KIFAP3	MIRT030958	hsa-miR-21-5p	B+
B++, S++, C++		MIRT037774	hsa-miR-671-3p	B++, S++, C++
		MIRT039305	hsa-miR-425-5p	B+, C
		ENST00000432442	GS1-519E5.1-001	
		ENST00000627551	RP11-573D15.8-018	
		ENST00000623027	CTA-992D9.11-001	
		ENST00000623959	RP3-323A16.1-001	
		ENST00000624300	CTD-2144E22.9-001	
ENSG00000233276	GPX1	MIRT040633	hsa-miR-92b-3p	B, C+

B+++, S++, C++		MIRT486916	hsa-miR-505-5p	B
		MIRT486917	hsa-miR-4537	B+, C
		MIRT486918	hsa-miR-1226-3p	B, C
		MIRT486919	hsa-miR-634	B+, S+, C
		ENST00000627551	RP11-573D15.8-018	
		ENST00000458252	AC123886.2-001	
		ENST00000527021	AP006621.9-001	
		ENST00000418602	RP11-536C5.7-001	
		ENST00000624790	RP11-572N21.1-001	
ENSG00000133103	COG6	MIRT630740	hsa-miR-875-5p	B+, C
B+, S++, C++		MIRT630741	hsa-miR-874-3p	B, C
		MIRT630742	hsa-miR-3157-3p	B+, S+, C
		MIRT630743	hsa-miR-4777-3p	B, C
		MIRT630744	hsa-miR-19b-2-5p	B
		ENST00000627551	RP11-573D15.8-018	
		ENST00000623111	MIR6820-001	
		ENST00000623959	RP3-323A16.1-001	
		ENST00000597346	KCNQ1OT1-001	
		ENST00000604411	TSIX-001	
ENSG00000153707	PTPRD	MIRT016634	hsa-miR-429	B+, C
B+, S+, C++		MIRT020345	hsa-miR-200a-3p	B+, S+,
		MIRT021067	hsa-miR-200c-3p	B
		MIRT021661	hsa-miR-141-3p	B+, C
		MIRT023978	hsa-miR-1-3p	B, S
		ENST00000627551	RP11-573D15.8-018	
		ENST00000527021	AP006621.9-001	
		ENST00000589281	RP11-95O2.5-001	
		ENST00000432442	GS1-519E5.1-001	
		ENST00000623959	RP3-323A16.1-001	

ENSG00000144724	PTPRG	MIRT020238	hsa-miR-130b-3p	B+, S+
B+, S++, C++		MIRT031199	hsa-miR-19b-3p	
		MIRT038215	hsa-miR-342-5p	B+
		MIRT039600	hsa-miR-624-5p	B+, S+, C+
		MIRT041395	hsa-miR-193b-3p	B, S
		ENST00000627551	RP11-573D15.8-018	
		ENST00000626826	HELLPAR-001	
		ENST00000623959	RP3-323A16.1-001	
		ENST00000623130	AC006548.28-001	
		ENST00000623111	MIR6820-001	
ENSG00000106278	PTPRZ1	MIRT022444	hsa-miR-124-3p	B
B+, S+, C++		MIRT025060	hsa-miR-181a-5p	B+, S+
		MIRT053463	hsa-miR-200c-3p	B
		ENST00000627551	RP11-573D15.8-018	
		ENST00000432442	GS1-519E5.1-001	
		ENST00000613502	RP4-737E23.2-002	
		ENST00000623959	RP3-323A16.1-001	
		ENST00000612722	FLJ16779-001	
ENSG00000132670	PTPRA	MIRT017376	hsa-miR-335-5p	
B++, S++, C++		ENST00000627551	RP11-573D15.8-018	
		ENST00000623959	RP3-323A16.1-001	
		ENST00000623111	MIR6820-001	
		ENST00000597346	KCNQ1OT1-001	
		ENST00000527021	AP006621.9-001	

B-whole blood, S- skeletal muscle, C-spinal cord,
(+++ strong expression; ++ moderate expression; + weak expression)
